# High cellphone use associated with greater risk of depression among young women aged 15–24 years in Soweto and Durban, South Africa

**DOI:** 10.1080/16549716.2021.1936792

**Published:** 2021-08-25

**Authors:** Janan J. Dietrich, Kennedy Otwombe, Tatiana E. Pakhomova, Keith J. Horvath, Stefanie Hornschuh, Khuthadzo Hlongwane, Kalysha Closson, Mamakiri Mulaudzi, Patricia Smith, Mags Beksinska, Glenda E. Gray, Mark Brockman, Jenni Smit, Angela Kaida

**Affiliations:** a Perinatal HIV Research Unit (PHRU), School of Clinical Medicine, Faculty of Health Sciences, University of the Witwatersrand, Johannesburg, South Africa; b African Social Sciences Unit of Research and Evaluation (ASSURE), School of Clinical Medicine, Faculty of Health Sciences, University of the Witwatersrand, Johannesburg, South Africa and Health Systems Research Unit, South African Medical Research Council, Cape Town, South Africa; c Faculty of Health Sciences, Simon Fraser University, Burnaby, Canada; d San Diego State University, Department of Psychology, San Diego, California, United States; e School of Population and Public Health, University of British Columbia, Vancouver, Canada; f MatCH Research Unit (MRU), Department of Gynaecology and Obstetrics, Faculty of Health Sciences, University of Witwatersrand, Johannesburg, South Africa; g Office of the President, South African Medical Research Council, Cape Town, South Africa

**Keywords:** Mental health, mobile health (mHealth), digital health, youth, South Africa

## Abstract

**Background:**

The ubiquity of cellular phone (cellphone) use in young people’s daily lives has emerged as a priority area of concern for youth mental health.

**Objective:**

This study measured the prevalence of depression and its association with high cellphone use among youth in Soweto and Durban, South Africa.

**Methods:**

We analysed cross-sectional, baseline survey data among youth aged 16–24 who participated in a dual-site cohort study, ‘AYAZAZI’, conducted from 2014 to 2017. The primary outcome was depression using the 10-item Center for Epidemiologic Studies Depression Scale, with a score of ≥ 10 indicating probable depression. Cellphone use was measured via self-reported average number of hours of active use, with ‘high cellphone use’ defined as daily usage of ≥ 8. Multivariable logistic regression models assessed the independent relationship between high cellphone use and probable depression, adjusting for potential confounders.

**Results:**

Of 425 participants with a median age of 19 years (IQR = 18–21), 59.5% were young women. Overall, 43.3% had probable depression, with a higher prevalence among women (49.0% vs. 34.9%, *P* = .004). Nearly all (94.6%) owned a cellphone. About one-third (29.5%) reported spending ≥ 8 hours per day using their cellphone (39.3% of women vs. 14.9% of men, *P* < .001). In the overall adjusted model, youth reporting high daily cellphone use had higher odds of probable depression (aOR: 1.83, 95% CI: 1.16–2.90). In gender-stratified models, high daily cellphone use was associated with probable depression among women (aOR: 2.51, 95% CI: 1.47–4.31), but not among men (aOR: 0.87, 95% CI: 0.35–2.16).

**Conclusions:**

Among a cohort of South African youth, we found a high prevalence of probable depression and high cellphone use (30%). The findings indicate a need for intersectoral initiatives focused on meaningful mental health support for South African youth to support positive growth and development.

## Background

Globally, mental health disorders account for a significant proportion of disease burden and disability among adolescents and young adults, and depression is associated with increased morbidity and suicide [1]. While most mental disorders tend to be diagnosed later in life, initial onset often occurs between 12 and 14 years of age, making adolescents and young adults a key population for mental health interventions [2]. In South African studies, reporting of significant depressive symptoms varies from 13.6% to 48% among youth [3–5]. Young women and sexual minority youth are at a disproportionately higher risk for mental health difficulties and psychosocial stress due to experiences of elevated discrimination and negative life events, including trauma and violence [6–9]. Among a sample of South African youth living with HIV, half had experienced eight or more adverse childhood events, including emotional abuse, physical abuse, sexual abuse, witnessing domestic violence, and witnessing community violence [10]. These multiple adverse events can be contextualised within a society that is still recovering from the effects of apartheid, with those from lower socio-economic settings most adversely affected [11]. Studies of South African youth show depression as a factor in increased HIV-risk behaviours [10], including reduced condom use [4], substance abuse [12] and transactional sex [3].

Globally, youth have rapidly integrated cellular phone (cellphone) use into their daily lives [13,14]. Currently, there is no universal definition for cellphone use. For the purposes of this study, cellphone use includes any measure of its use, normative or problematic, that quantifies the extent to which a person uses a phone or demonstrates dependency, irrespective of the purpose for which the phone is used [15]. In South Africa, cellphone use among young people, particularly smartphone use, has increased dramatically over the past decade and has emerged as an important area for youth health [16]. As cellphone capabilities continue to grow, allowing for constant connectivity through online communication, there has been heightened awareness of the possible connections between technology use and mental health [13,17–19]. However, cellphone use among youth in South Africa depends on their resources, including socio-economic status, cellphone ownership, and access to data or Wi-Fi [20]. Youth living in lower socio-economic settings may be more vulnerable to crime, including home and street robberies (having their phone stolen), and poorer overall access to basic needs [21,22].

Reporting of the relationship between social media use and poor mental health outcomes has been inconsistent, with no data available from South Africa. A study by Hoare et al. investigated the relationship between social media use among Australian adolescents and showed that spending ≥ 7 hours a day using the internet was associated with depression in females and psychological distress in males [18]. Although almost exclusively from Western countries and Asia, some studies have found associations between Facebook use and mental health outcomes, depression, increased stress, and lower self-esteem [23,24]. Other studies have found no associations or positive effects of Facebook use, particularly as a means of increased social connectivity, social capital growth, and increased interaction for individuals with lower self-esteem [25–28]. These data indicate that increased time using the internet is an area still requiring investigation to understand high-frequency use when compared with excessive and problematic use.

Researchers distinguish between excessive and problematic cellphone use [28–31]. Although there are at least 78 existing validated scales that measure, identify and characterise excessive or problematic smartphone use, there are currently no established standardised cut-offs to define high and/or excessive cellphone use [32]. However, factors such as age and gender play an important role in predicting excessive cellphone use and problematic online behaviour [28]. In global studies investigating the association between technology use and mental health, results have shown that young women, who tend to engage more in social media across all age groups, have been shown to have higher rates of depression. For men, higher rates of excessive cellphone use are related to internet addiction, gaming, and accessing sexually gratifying content [29–31].

Several studies have found associations between depression, anxiety and psychological distress with time spent on the internet or cellphone ranging from one to nine or more hours per day [18,23,33–36]. A Swiss study assessing problematic cellphone use, (determined by tracking messaging volume and data traffic), found that high frequency of use associated with smartphone ownership was also linked with impaired psychological well-being, depression, and other behavioural problems [37]. A recent review of the literature on excessive smartphone use found that compulsive use of cellphones shares similarities with problematic use of other technologies, including psychological dependence, maladaptive coping, and negative mental health consequences [38]. Research on excessive and problematic cellphone use in sub-Saharan Africa and its consequences is still in its infancy, and insights into this field are limited.

To date, research on cellphone use in the South African context has focused primarily on technology uptake and cellphone-based HIV-related interventions, including their use for study recruitment, health promotion, and HIV care and prevention [39–41]. A few South African studies have investigated the use of text messages to improve adherence to mental health services [42] as well as adherence to psychotropic medication [43]. In the current global crisis of COVID-19, acquiring a better understanding of mental health and providing virtual alternatives for interventions have become crucial. Little work currently exists assessing high cellphone use and its connection to mental health outcomes among young South Africans. A 2014 study by North et al. showed some evidence of high cellphone use by South African university students and tendencies towards cellphone addiction [44]. A South African study assessing e-media use and neurocognitive outcomes was conducted among younger adolescents (9–16 years) [45]. However, more research is needed to fully understand the possible associations between cellphone use and mental health, especially among youth. There are marked disparities between existing South African studies on the relationship between cellphone use and mental health outcomes. Research conducted among youth in Soweto found differences by gender in terms of using social networking and health risks, with females reporting higher use of social networking [39,46–48]. Given these gaps, the objective of this study was to measure the prevalence of depression and its independent association with high cellphone use among South African youth, overall and by gender.

## Methods

### Study design and setting

The name ‘AYAZAZI’ is derived from Zulu (one of South Africa’s 11 official languages) – ‘aya’ meaning adolescents and young adults and ‘zazi’ meaning knowing themselves. AYAZAZI was a prospective longitudinal cohort study from November 2014 to April 2017, aimed at linking socio-behavioural, clinical and biomedical data from youth aged 16–24 years. Participants were recruited from Soweto and Durban, South Africa, and were followed for a total of 18 and 12 months, respectively, with scheduled visits every six months. Data from the baseline visit were used for the present analysis.

Soweto and Durban are densely populated South African cities in which most youth have access to cellphones [39,46], but experience high rates of unemployment and poor socio-economic status, including high food insecurity [21,49]. Previous work in Soweto indicates a high incidence of depression [4,5,7,50]. The Soweto cohort was based at the Perinatal HIV Research Unit (PHRU) located at Chris Hani Baragwanath Hospital. The Durban cohort was based at the Commercial City research site led by the MatCH Research Unit (MRU). Study inclusion criteria were 16–24 years of age, residing in Soweto or Durban, self-reporting an HIV-negative or unknown HIV status, and being willing and able to provide voluntary written informed consent for study procedures. Exclusion criteria included current participation in another clinical or observational HIV prevention study, lack of parental consent for candidates below 18 years, and an inability to provide informed consent. At both sites, a community outreach strategy was used to recruit participants, by using posters, pamphlets and word-of-mouth. In Soweto, participants were also recruited through the PHRU’s HIV Counselling and Testing Clinic and in Durban, participants were also approached through a nearby public sector reproductive health clinic. Additional details about the study have been published elsewhere [51].

### Measures

#### Questionnaire

Trained youth interviewers administered a structured online questionnaire (supported by DataFAX^TM^ software) at baseline. Questionnaires were delivered in English, isiZulu, or Sesotho in Soweto and in English or isiZulu in Durban, according to each participant’s preference. Questionnaires were designed in English with input from the adolescent community advisory boards. Thereafter, questionnaires were translated into isiZulu and Sesotho by a local translation company and reviewed by the adolescent community advisory boards. Questionnaires took approximately 60 minutes to complete.

#### Main outcome

*Depression* was assessed using the 10-item Center for Epidemiologic Studies Short Depression Scale (CES-D-10) [52], a depression screening tool validated in South Africa [53], and demonstrated an acceptable Cronbach’s alpha score (α = 0.71) in our study sample [54]. Each item assesses a dimension of mood over the past seven days with a four-point Likert response scale (less than a day, 1–2 days, 3–4 days, 5–7 days). Probable depression is defined as a cut-off score of ≥ 10 [53].

#### Main exposure

The exposure of interest was high cellphone usage. We asked participants whether they owned a cellphone (yes vs. no) and, if they answered yes, what activities they used it for. Participants could select from the multiple options: general communication (including sending and receiving SMSes); making phone calls and e-mail; mobile entertainment (including listening to music/radio, playing games, and accessing the internet and YouTube; Mxit, Facebook, Twitter, WhatsApp, BBM, We Chat, Instagram, 2Go); for emergencies only; cellphone banking, and an ‘other’ option. We then asked participants, ‘*How much time in a day do you spend actively using a cellphone? This includes listening to music/radio, SMS, making phone calls, playing games and accessing the internet*.’ Response options were: 0–1 hours, 2–4 hours, 5–7 hours and ≥8 hours per day. ‘High cellphone use’ was based on these categories already used in the survey and defined as active daily usage ≥8 hours in comparison with <8 hours. Participants were asked additional questions about their cellphone use, including: ‘*How do you get airtime? (prepaid and contract)’*; and *‘Who pays for the prepaid/contract airtime?’*

Internet access and usage were assessed through the following questions, which were analysed separately: ‘*In the past 6 months, have you had access to the internet?*’ and ‘*When you are on the internet, what do you do or search for?*’ For the latter question, participants selected all options given: downloading music and videos, research for school projects, looking up health information, looking up information about sex, apps, dating sites, getting directions to places (GPS – global positioning system) and looking at maps, finding out about parties/DJ events, internet banking, using social media sites, software, e-mail, selling and buying sites, and other.

These variables were: selected based on known relationships with depression and cellphone use [9,39,46,49]. study site (Soweto or Durban); self-identified gender (man, woman or other); sexual orientation (heterosexual, lesbian, gay, bisexual, transgender, queer or questioning); housing (formal [brick house, townhouse, flat/apartment] vs, reconstruction development program (RDP) house, shack [informal settlement], or shack [backyard]); female-headed household (yes vs. no); median quartile 1–quartile 3(Q1–Q3) number of adults in the home; median Q1–Q3 number of children in the home; income in the past 3 months (1–400 ZAR, 401–800 ZAR, 801–1 600 ZAR, 1 601–3 200 ZAR, ≥3 201 ZAR); number of people financially dependent on participant (none vs. ≥1); currently enrolled in any school (no vs. yes), and had ever had consensual sexual intercourse, which included oral, vaginal and/or anal sex; consensual sex was defined as both partners having agreed to have sexual intercourse.

### Statistical analyses

We first used descriptive statistics to summarise the sample. Continuous measures were summarised descriptively using the medians, Q1-Q3 already defined. Frequencies and percentages were determined for categorical variables. Next, we assessed the associations between depressive symptoms and socio-demographics and cellphone use, stratified by gender. Both continuous and categorical variables were stratified by gender and symptoms of probable depression (yes vs. no). To test for statistical significance for categorical measures stratified by gender and probable depression, Pearson’s chi-square analysis or Fisher’s exact test was used. The medians and interquartile range (IQR) were compared between groups using the Kruskal-Wallis test.

We dichotomised the exposure variable into high (≥8 hours) and low (<8 hours). Using crude and adjusted logistic regression analyses overall and by gender, we assessed the independent association between high cellphone use and depressive symptoms, controlling for potential confounding. Variables with a *p*-value ≤ 0.2 at univariate level thought to be explanatory variables based on *a priori* knowledge were included in the full multivariable model. Thereafter, a backward selection procedure was used to determine the final model with the lowest Akaike information criterion (AIC). The Hosmer-Lemeshow statistic was used to measure model diagnostics/fit in the final multivariate model. Regression results are presented as Odds Ratio (OR) in the univariate and adjusted OR (aOR) in the final multivariate model. All statistical analysis was conducted in SAS Enterprise Guide 7.1 (SAS Institute, Cary, NC) using SAS/STAT procedures PROC FREQ, PROC NPAR1WAY and PROC LOGISTIC.

### Ethical considerations

The AYAZAZI study was approved by the University of the Witwatersrand, Johannesburg, South Africa, and Simon Fraser University, Burnaby, Canada. Written informed consent was obtained for participants 18 years and older. Written assent was obtained from minors younger than 18 years, together with written informed consent from a parent or legal guardian. Participants received a 150 ZAR (~$12 USD) reimbursement per scheduled visit to compensate for transportation and time.

## Results

### Demographic characteristics by gender

Of the 425 participants included in this analysis, 59.3% (n = 253) were women (Table 1). Most participants were 16–20 years old (74.1%, n = 315/425), heterosexual (93.2%, n = 395/424), living in formal housing (70.8%, n = 301/425), receiving an average income of less than 1600ZAR (77.2%, n = 328/425), currently students (71.5%, n = 303/424). Most had no dependents (71.3%, n = 303/425) and the majority had had sex in their lifetime (85.1%, n = 361/424).

**Table 1.** Baseline characteristics among male and female participants of AYAZAZI in South Africa

**Variables**	**Overall (n = 425)**	**Young women** **(n = 253)**	**Young men (n = 172)**	***P*-value**
**Site**				
Durban (%)	220 (51.8)	132 (52.2)	88 (51.2)	0.84
Soweto (%)	205 (48.2)	121 (47.8)	84 (48.8)	
**Age group (in years)**				
16–20 (%)	315 (74.1)	189 (74.7)	126 (73.3)	0.74
21–24 (%)	110 (25.9)	64 (25.3)	46 (26.7)	
**Median age [IQR]**	19.0 [18.0–21]	19.0 [17.0–21]	19.0 [18.0–21]	0.77
**Sexual orientation** ^a^				
Heterosexual (%)	395 (93.2)	235 (92.9)	160 (93.6)	0.79
LGBTQl (%)	29 (6.8)	18 (7.1)	11 (6.4)	
**Main home language**				
IsiZulu (%)	310 (72.9)	181 (71.5)	129 (75.0)	0.43
Other (%)	115 (27.1)	72 (28.5)	43 (25.0)	
**Type of housing**				
Formal housing (%)	301 (70.8)	165 (65.2)	136 (79.1)	0.*002*
Other (%)	124 (29.2)	88 (34.8)	36 (20.9)	
**Head of household** ^b^				
Female (%)	259 (60.9)	164 (64.8)	95 (55.2)	0.*047*
Other (%)	166 (39.1)	89 (35.2)	77 (44.8)	
**Monthly personal income (ZAR)**				
None–1600 (%)	328 (77.2)	204 (80.6)	124 (72.1)	0.*04*
1601–More (%)	97 (22.8)	49 (19.4)	48 (27.9)	
**Median number of adults in the household (IQR)**	3.0 (2.0–4.0)	3.0 (2.0–4.0)	3.0 (2.0–4.0)	0.49
**Median number of children in the household (IQR)**	2.0 (0–3.0)	2.0 (1.0–3.0)	1.0 (0.0–2.0)	0.*003*
**Currently a student?**				
No (%)	121 (28.5)	71 (28.2)	50 (29.1)	0.84
Yes (%)	303 (71.5)	181 (71.8)	122 (70.9)	
**Number of persons financially depending on participant**				
0 (%)	303 (71.3)	174 (68.8)	129 (75.0)	0.16
≥1 (%)	122 (28.7)	79 (31.2)	43 (25.0)	
**Ever had consensual sex**				
No (%)	63 (14.9)	46 (18.2)	17 (9.9)	0.*02*
Yes (%)	361 (85.1)	207 (81.8)	154 (90.1)	

*P*-values that are italicized are statistically significant at the 0.05 level.

^a^LGBTQ: Lesbian, gay, bisexual, transgender, queer or questioning

^b^Other is defined as Male adult, Male child, Male child <18 years, more than 1 person, Me (participant), and don’t know

Relative to men, women were more likely to receive an average income of ≤1600ZAR (80.6% vs. 72.1%; *P* = .04). Conversely, men were more likely than women to live in formal housing (79.1% vs. 65.2%; *P* = .002) and have had consensual (oral, vaginal or anal) sex (90.1% vs. 81.8%; *P* = .02).

### Cellphone use

Of the 425 participants, 94.6% (n = 402) owned a cellphone. Those with access used cellphones for general communication (97.2%, n = 410/422), mobile entertainment (91.9%, n = 388/422), WhatsApp (77.5%, n = 327/422), Facebook (71.1%, n = 300/422), Twitter (18.7%, n = 79/422) and Instagram (13.7%, n = 58/422). Most received airtime on prepaid (97.6%, n = 412/422) and paid for their own airtime (71.2%, n = 292/410). A significantly higher proportion of women than men owned a cellphone (96.4% vs. 91.9%; *P* = .04) and spent ≥8 hours using their cellphone daily (39.3% vs. 14.9%; *P* < .001). In the preceding six months all participants had used their cellphones primarily to search for apps on the internet (62.3% vs. 40.6%; *P* < .001) (Table 2). Relative to women, men were more likely to use Twitter (24.7% vs. 14.7%; *P* = .01), Instagram (18.8% vs. 10.3%; *P* = .01), to pay for their own prepaid airtime (82.2% vs. 64.0%; *P* < .001), download music and videos (71.2% vs. 36.9%; *P* < .001) and search for information about sex using their phones (17.7% vs. 8.3%; *P* = .004).

**Table 2.** Cellphone usage among young male and female participants of AYAZAZI

**Variables**	**Overall (n = 425)**	**Young women** **(n = 253)**	**Young men (n = 172)**	***P*-value**
**Do you have your own cellphone?**				
No (%)	23 (5.4)	9 (3.6)	14 (8.1)	0.*04*
Yes (%)	402 (94.6)	244 (96.4)	158 (91.9)	
**What do you use the cellphone for?**				
General communication (%)	410 (97.2)	248 (98.4)	162 (95.3)	0.06
Mobile entertainment (%)	388 (91.9)	235 (93.3)	153 (90.0)	0.22
Facebook (%)	300 (71.1)	184 (73.0)	116 (68.2)	0.29
Twitter (%)	79 (18.7)	37 (14.7)	42 (24.7)	0.*01*
WhatsApp (%)	327 (77.5)	200 (79.4)	127 (74.7)	0.26
Instagram (%)	58 (13.7)	26 (10.3)	32 (18.8)	0.*01*
**Amount of time in a day spent actively using a cellphone**				
< 8 Hours (%)	296 (70.5)	153 (60.7)	143 (85.1)	*<0.001*
≥ 8 Hours (%)	124 (29.5)	99 (39.3)	25 (14.9)	
**How do you get airtime?**				
Contract (%)	10 (2.4)	5 (2.0)	5 (2.9)	0.53
Prepaid (%)	412 (97.6)	247 (98.0)	165 (97.1)	
**Who pays for the prepaid?**				
Participant (%)	292 (71.2)	158 (64.0)	134 (82.2)	*<0.001*
Other (%)	118 (28.8)	89 (36.0)	29 (17.8)	
**Do you have access to the internet?**				
No (%)	45 (10.6)	28 (11.1)	17 (9.9)	0.76
Yes (%)	380 (89.4)	225 (88.9)	155 (90.1)	
**When you are on the internet what do you do or search for?**				
Download music and videos (%)	214 (50.7)	93 (36.9)	121 (71.2)	*<0.001*
Research for school projects (%)	247 (58.5)	141 (56.0)	106 (62.4)	0.19
Look up information about health (%)	97 (23.0)	51 (20.2)	46 (27.1)	0.10
Search for information about sex (%)	51 (12.1)	21 (8.3)	30 (17.7)	0.*004*
Apps (%)	226 (53.6)	157 (62.3)	69 (40.6)	*<0.001*

*P*-values that are italicized are statistically significant at the 0.05 level.

### Depression by demographic characteristics and use of technology

Just under half (43.3%; n = 184/425) had probable depression based on their CES-D-10 scores. Women were more likely to have probable depression than men (49.0% vs. 34.9%; *P* = .004). Youth with probable depression were likely to: spend ≥ 8 hours using a cellphone daily (55.7% vs. 38.2%, *P* = .001); receive an average income of more than 1600ZAR (55.7% vs. 39.6%; *P* = .005); have at least one dependent (54.9% vs. 38.6%; *P* = .002); and search for apps in the past 6 months (48.2% vs. 37.2%; *P* = .02) (Table 3). A higher proportion of youth with probable depression reported the use of Instagram than youth who did not have probable depression (45.1% vs. 31.0%, *P* = .045).

**Table 3.** Depression by demographic and cellphone use co-variates

**Variables**	**Overall** **(n = 425)**	**Probable depression(n = 184)**	**Not Depressed(n = 241)**	***P* value**
**Site**				
Durban (%)	220 (51.8)	95 (43.2)	125 (56.8)	0.96
Soweto (%)	205 (48.2)	89 (43.4)	116 (56.6)	
**Gender**				
Women (%)	253 (59.5)	124 (49.0)	129 (51.0)	0.*004*
Men (%)	172 (40.5)	60 (34.9)	112 (65.1)	
**Age group (in years)**				
16–20 (%)	315 (74.1)	133 (42.2)	182 (57.8)	0.45
21–24 (%)	110 (25.9)	51 (46.4)	59 (53.6)	
**Sexual orientation** ^c^				
Heterosexual (%)	395 (93.2)	169 (42.8)	226 (57.2)	0.35
LGBTQ (%)	29 (6.8)	15 (51.7)	14 (48.3)	
**Main home language**				
IsiZulu (%)	310 (72.9)	134 (43.2)	176 (56.8)	0.96
Other (%)	115 (27.1)	50 (43.5)	65 (56.5)	
**Type of housing**				
Formal Housing (%)	301 (70.8)	129 (42.9)	172 (57.1)	0.78
Other (%)	124 (29.2)	55 (44.4)	69 (55.6)	
**Head of household**				
Female (%)	259 (60.9)	115 (44.4)	144 (55.6)	0.56
Other (%)	166 (39.1)	69 (41.6)	97 (58.4)	
**Average own income (In Rand)**				
None–1600 (%)	328 (77.2)	130 (39.6)	198 (60.4)	0.*005*
1601–More (%)	97 (22.8)	54 (55.7)	43 (44.3)	
**Currently a student?**				
No (%)	121 (28.5)	61 (50.4)	60 (49.6)	0.06
Yes (%)	303 (71.5)	122 (40.3)	181 (59.7)	
**Number of persons financially depending on participant**				
<1 (%)	303 (71.3)	117 (38.6)	186 (61.4)	0.*002*
≥1 (%)	122 (28.7)	67 (54.9)	55 (45.1)	
**Ever had consensual sex**				
No (%)	63 (14.9)	25 (39.7)	38 (60.3)	0.52
Yes (%)	361 (85.1)	159 (44.0)	202 (56.0)	
**Do you have your own cellphone?**				
No (%)	23 (5.4)	7 (30.4)	16 (69.6)	0.20
Yes (%)	402 (94.6)	177 (44.0)	225 (56.0)	
**What do you use the cellphone for:**				
General Communication (%)	410 (97.2)	178 (43.4)	232 (56.6)	0.49
Mobile Entertainment (%)	388 (91.9)	172 (44.3)	216 (55.7)	0.09
Facebook (%)	300 (71.1)	135 (45.0)	165 (55.0)	0.22
Twitter (%)	79 (18.7)	34 (43.0)	45 (57.0)	0.99
WhatsApp (%)	327 (77.5)	145 (44.3)	182 (55.7)	0.35
Instagram (%)	58 (13.7)	18 (31.0)	40 (69.0)	0.*045*
**Amount of time in a day spent actively using a cellphone**				
< 8 Hours (%)	296 (70.5)	113 (38.2)	183 (61.8)	0.*001*
≥ 8 Hours (%)	124 (29.5)	69 (55.7)	55 (44.3)	
**How do you get airtime?**				
Contract (%)	10 (2.4)	6 (60.0)	4 (40.0)	0.28
Prepaid (%)	412 (97.6)	176 (42.7)	236 (57.3)	
**Who pays for the prepaid?**				
Participant (%)	292 (71.2)	121 (41.4)	171 (58.6)	0.42
Other (%)	118 (28.8)	54 (45.8)	64 (54.2)	
**Do you have access to the internet?**				
No (%)	45 (10.6)	19 (42.2)	26 (57.8)	0.88
Yes (%)	380 (89.4)	165 (43.2)	215 (56.8)	
**When you are on the internet what do you do or search for?**				
Download music and videos (%)	214 (50.7)	86 (40.2)	128 (59.8)	0.22
Research for school projects (%)	247 (58.5)	104 (42.1)	143 (57.9)	0.61
To find information about health (%)	97 (23.0)	37 (38.1)	60 (61.9)	0.26
To find information about sex (%)	51 (12.1)	24 (47.1)	27 (52.9)	0.55
Apps (%)	226 (53.6)	109 (48.2)	117 (51.8)	0.*02*

^a^LGBTQ: Lesbian, gay, bisexual, transgender, queer or questioning

### Overall logistic regression model for probable depression

In the multivariate model (Table 4), spending ≥ 8 hours using a cellphone daily (aOR: 1.83, 95% CI: 1.159–2.90; *P* = .01) was independently associated with probable depression. In addition, probable depression was significantly associated with: being a woman (aOR: 1.591, 95% CI: 1.032–2.454; *P* = .04), earning an average income of at least 1601ZAR (aOR: 1.726, 95% CI: 1.046–2.849, *P* = .03) and having one or more financial dependents (aOR: 1.712, 95% CI: 1.070–2.741; *P* = .03). Model fit was assessed using the Hosmer-Lemeshow goodness-of-fit statistic that indicated a good fit (*P* = 0.51).

**Table 4.** Unadjusted and adjusted odds ratios (95% confidence intervals (CI)) of variables associated with probable depression among young men and women overall, South Africa (n = 419)

	Unadjusted		Adjusted	
Variables	OR 95% (CI)	P-value	OR 95% (CI)	P-value
**Primary exposure**				
**Amount of time in a day spent actively using a cellphone**				
≥ 8 hours vs < 8 hours	2.03 (1.33–3.11)	0.01	*1.83 (1.16–2.90)*	0.*01*
**Potential Confounders**				
**Site**				
Soweto vs Durban	1.01 (0.69–1.48)	0.96	-	-
**Gender**				
Women vs. Men	1.79 (1.20–2.67)	0.004	*1.59 (1.03*–*2.45)*	0.*04*
**Age group (in years)**				
21–24 vs 16–20	1.18 (0.77–1.83)	0.45	-	-
**Sexual orientation** ^d^				
LGBTQ vs heterosexual	1.43 (0.67–3.05)	0.35	-	-
**Type of housing**				
Other vs formal housing	1.06 (0.70–1.62)	0.78	-	-
**Head of household**				
Female vs. Other	1.12 (0.76–1.67)	0.57	-	-
**Average own income (in Rand)**				
1601–More vs None–1600	1.91 (1.21–3.02)	0.006	*1.73 (1.05–2.85)*	0.*03*
**Are you a student**				
No vs Yes	1.51 (0.99–2.3)	0.06	1.09 (0.68–1.74)	0.72
**Number of persons financially depending on participant**				
≥1 vs 0	1.94 (1.27–2.96)	0.002	*1.71 (1.07–2.74)*	0.*03*
**Ever had consensual sex**				
Yes vs No	1.25 (0.69–2.07)	0.52	-	-
**Do you have your own cell phone?**				
Yes vs No	1.88 (0.72–4.47)	0.21	1.78 (0.58–5.80)	0.33

^d^LGBTQ: Lesbian, gay, bisexual, transgender, queer or questioning. The italicized ORs are significant as they do not contain the null.

### Logistic regression models by gender for probable depression

In young women, probable depression was associated with spending ≥ 8 hours using a cellphone daily (aOR: 2.514, 95% CI: 1.465–4.313; *P* = .001), earning an average of more than 1601ZAR (aOR: 2.213, 95% CI: 1.108–4.420; *P* = .02) and having at least one financial dependent (aOR: 1.893, 95% CI: 1.046–3.426; *P* = .03). Model fit for women suggested a good fit under the Hosmer-Lemeshow statistics (*P* = .96) (Table 5). Unadjusted and adjusted results for men found no significant associations.

**Table 5.** Crude and adjusted logistic regression models examining associations between high cellphone use and depressive symptoms among young women and young men participating in baseline AYAZAZI survey (n = 420)

	Women (n = 252)				Men (n = 168)			
	Univariate		Multivariate		Univariate		Multivariate	
Variables	OR 95% (CI)	*P*-value	OR 95% (CI)	*P*-value	Adjusted OR 95% (CI)	*P*-value	Adjusted OR 95% (CI)	*P*-value
**Amount of time in a day spent actively using a cell phone**								
< 8 hours	(ref)	-	-	-	(ref)	-	(ref)	-
≥ 8 hours	2.29 (1.37–3.85)	.002	2.51 (1.45–4.31)	.*001*	0.88 (0.35–2.17)	.77	0.87 (0.35–2.16)	0.76
**Potential confounders**								
**Site**								
Durban	1.02 (0.62–1.67)	.94	-	-	0.93 (0.50–1.74)	.82	-	-
Soweto	(ref)	-	-	-	(ref)		-	-
**Age group (in years)**								
16–20	(ref)	-	-	-	(ref)	-	-	-
21–24	1.25 (0.71–2.20)	.45	-	-	1.13 (0.56–2.29)	.73	-	-
**Sexual orientation** ^e^								
LGBTQ	1.70 (0.64–4.53)	.29	-	-	1.06 (0.30–3.78)	.93	-	-
Heterosexual	(ref)	-	-	-	(ref)	-	-	-
**Type of housing**								
Formal housing	1.16 (0.69–1.95)	.57	-	-	0.80 (0.377–1.72)	.57	-	-
Other	(ref)	-	-	-	(ref)	-	-	-
**Head of household**								
Female	0.91 (0.54–1.52)	.72	-	-	1.35 (0.71–2.55)	.36	-	-
Other	(ref)	-	-	-	(ref)	-	-	-
**Average own income (In Rand)**								
None–1600	(ref)	-	-	-	(ref)	-	-	-
1601–More	2.56 (1.33–4.94)	.005	2.21 (1.11–4.42)	.*02*	1.70 (0.86–3.36)	.13	1.39 (0.66–2.94)	0.39
**Are you a student**								
No	2.29 (1.30–4.02)	.004	-	-	0.83 (0.41–1.68)	.61	-	-
Yes	(ref)	-	-	-	(ref)	-	-	-
**Number of persons financially depending on participant**								
<1	(ref)	-	-	-	(ref)	-	-	-
≥1	2.00 (1.16–3.43)	.01	1.89 (1.05–3.43)	.*03*	1.70 (0.84–3.45)	.14	1.48 (0.68–3.2)	0.33
**Ever had consensual sex**								
No	(ref)	-	-	-	(ref)	-	-	-
Yes	1.83 (0.95–3.5)	.07	1.31 (0.64–2.67)	.45	0.57 (0.21–1.57)	.28	-	-
**Do you have your own cell phone?**								
No	(ref)	-	-	-	(ref)	-	-	-
Yes	8.13 (1.00–66.01)	.049	-	-	0.69 (0.23–2.10)	.52	-	-

^e^LGBTQ: Lesbian, gay, bisexual, transgender, queer or questioning.

*P*-values that are italicized are statistically significant at the 0.05 level.

## Discussion

Our study examined the relationship between cellphone use and probable depression among South African youth. Of concern is that nearly half of the sample met the CES-D-10 criteria for having significant depressive symptoms or probable depression, with a higher prevalence of probable depression among young women than young men. Cellphone ownership and high cellphone use were significantly higher in young women. High cellphone use was independently associated with probable depression among women but not among men.

Our study contributes to a better understanding of depression post-apartheid in marginalised youth [55]. However, a comparison with previous South African studies on depression in youth is complex as different scales are used to measure depression; even when using the CESD-10, studies may use different cut-off scores [3]. Nonetheless, our results confirm a high proportion of youth with probable depression, which corroborates the results of previous research among adults that reported that living in an adverse (poorer) neighbourhood and being unemployed were independently associated with depression as measured by the CES-D-10 [56].

Nearly all participants in our study owned a cellphone, which is consistent with national cellphone ownership data among adults in South Africa [57] and previous research with youth in Soweto [46]. The continuously rising cellphone penetration rates among South African youth, including increased access and use of smartphones [58], provides a promising entryway for delivering mental health interventions tailored to individual needs. Our data suggest that technology-based depression interventions should be prioritised for young women since they seem to exhibit higher levels of depression and use their cellphones throughout the day. The findings are consistent with previous research on depression and cellphone use among youth in Soweto [4,39,46]. Cellphone-based health interventions show promise in improving public health outcomes [59,60], but it is still unclear how these are implemented. Their effectiveness among young populations in resource-limited settings such as South Africa requires investigation. The findings from this study provide an opportunity for future directed research in designing a cellphone-based mental health intervention for South African youth.

We did not find a direct association between general social network usage and probable depression in participants. In bivariate analyses, young people who used Instagram were less likely to be depressed or it could be that those with a higher socio-economic status were able to download and use Instagram. A 2015 study by Lup and Rosenthal found that although Instagram was associated with depression, the relationship with depression was tied to social comparison and following strangers, whereas positive associations were observed when individuals followed family and friends [61]. Although we did not collect data on the nature of social media usage, there are likely to be multiple contributing factors which influence negative experiences with technology, and there is some evidence that personality constructs, gender, self-esteem, and social behaviours play an important role in determining problematic and excessive social media usage [27,28].

Our study found that participants with probable depression were less likely to use their phones to download and use apps. In addition, socio-demographic factors may influence the association between high cellphone use and mental health and may be applicable only to South African youth who can afford to pay for enough data to download apps and spend extended periods on their phones. In South African adults, depression is directly associated with limited material wealth [62]. Our data indicate that higher income is associated with poorer mental health outcomes. However, within the context of this study, higher income was still below a living wage in South Africa [63]. We can assume that participants did not have access to sufficient financial resources to meet their basic needs. Unfortunately, income is a difficult variable to modify, and youth may have had even more data restrictions and loss of income due to the impact of the COVID-19 pandemic and stay-at-home measures. Certainly, a more nuanced understanding is required to explore how cellphone use, particularly smartphone functionalities, could be leveraged for mental health support in youth, and in particular during times where stay-at-home orders are recommended.

### Limitations

A limitation of this paper is that we did not include a validated scale to measure high cellphone usage, indicating a need to investigate the threshold at which cellphone is use is deemed excessive in South African populations. As the analysis used cross-sectional data, the direction of effect between depression and excessive cellphone use cannot be determined. However, our regression analysis does provide some guidance about the direction that we investigated. For our study, the dependent or exposure variable was frequency of daily cellphone use.

Having surveyed individuals in a predominantly lower socio-economic area who probably experienced limitations in data access also affected our assessment of excessive usage, including social media, and thus may not be representative of the general population. However, nearly one-third of participants reported using their phones for ≥8 hours a day indicating some accessibility to cellphone usage despite socio-economic status. Future research should explore the association between increased screen time as well as problematic use, resilience and coping, as a means to better understand the high levels of dependency on cellphone technology in young South Africans.

## Conclusion

Our study found high prevalence of depressive symptoms and high cellphone use in youth, with high cellphone use associated with probable depression in women but not in men. The high proportion of participants reporting significant symptoms of depression indicates a need for intersectoral initiatives focused on meaningful mental health support for youth living in vulnerable communities, to support positive growth and development.

## Paper context

This research found high prevalence of probable depression and high cellphone use in a youth cohort from Soweto and Durban, South Africa, with high cellphone use associated with probable depression in women but not in men. The high proportion of participants reporting significant symptoms of depression indicates a need for public health initiatives focused on meaningful mental health support for youth living in vulnerable communities, to support positive growth and development.

## References

[1] Zhang WC, Jia CX, Zhang JY, et al. Negative life events and attempted suicide in rural China. PLOS One. 2015;10:1–12. 10.1371/journal.pone.0116634 PMC4303417 25611854

[2] Patel V, Flisher AJ, Hetrick S, et al. Mental health of young people: a global public-health challenge. Lancet. 2007;369:1302–1313. 17434406 10.1016/S0140-6736(07)60368-7

[3] Nduna M, Jewkes RK, Dunkle KL, et al. Associations between depressive symptoms, sexual behaviour and relationship characteristics: a prospective cohort study of young women and men in the Eastern Cape, South Africa. JAIDS. 2010;13:1–8. 10.1186/1758-2652-13-44 PMC2992477 21078150

[4] Barhafumwa B, Dietrich J, Closson K, et al. High prevalence of depression symptomology among adolescents in Soweto, South Africa associated with being female and cofactors relating to HIV transmission. Vulnerable Child Youth Stud. 2016;11:263–273.

[5] Otwombe K, Dietrich J, Laher F, et al. Health-seeking behaviours by gender among adolescents in Soweto, South Africa. Glob Health Action. 2015;8:1–9. 10.3402/gha.v8.25670 PMC4315777 25653113

[6] Tomlinson M, Grimsrud AT, Stein DJ, et al. The epidemiology of major depression in South Africa: results from the South African stress and health study. S Afr Med J. 2009;99:367–373. 19588800 PMC3195337

[7] Closson K, Dietrich JJ, Nkala B, et al. Prevalence, type, and correlates of trauma exposure among adolescent men and women in Soweto, South Africa: implications for HIV prevention. BMC Public Health. 2016;16:1191. 27884181 10.1186/s12889-016-3832-0 PMC5123224

[8] Mutumba M, Harper GW. Mental health and support among young key populations: an ecological approach to understanding and intervention. JAIDS. 2015;18:19429. 10.7448/IAS.18.2.19429 PMC4344542 25724505

[9] Thurston IB, Dietrich J, Bogart LM, et al. Correlates of sexual risk among sexual minority and heterosexual South African youths. Am J Public Health. 2014;104:1265–1269. 24832149 10.2105/AJPH.2013.301865 PMC4056197

[10] Kidman R, Nachman S, Dietrich J, et al. Childhood adversity increases the risk of onward transmission from perinatal HIV-infected adolescents and youth in South Africa. Child Abuse Negl. 2018;79:98–106. 29428881 10.1016/j.chiabu.2018.01.028 PMC5878998

[11] Jewkes R. Intimate partner violence: causes and prevention. Lancet. 2002;359:1423–1429. 11978358 10.1016/S0140-6736(02)08357-5

[12] Saban A, Flisher AJ, Grimsrud A, et al. The association between substance use and common mental disorders in young adults: results from the South African Stress and Health (SASH) survey. Pan Afr Med J. 2014;17:1–7. 10.11694/pamj.supp.2014.17.1.3328 PMC3946226 24624244

[13] Lin LY, Sidani JE, Shensa A, et al. Association between social media use and depression among US young adults. Depress Anxiety. 2016;33:323–331. 26783723 10.1002/da.22466 PMC4853817

[14] Vacaru M, Shepherd R, Sheridan J. New Zealand youth and their relationships with mobile phone technology. Int J Ment Health and Addiction. 2014;12:572–584.

[15] Kates AW, Wu H, Coryn CL. The effects of mobile phone use on academic performance: a meta-analysis. Comput Educ. 2018;127:107–112.

[16] Abi-Jaoude E, Naylor KT, Pignatiello A. Smartphones, social media use and youth mental health. CMAJ. 2020;192:e136–e141. 32041697 10.1503/cmaj.190434 PMC7012622

[17] Boulos MNK, Brewer AC, Karimkhani C, et al. Mobile medical and health apps: state of the art, concerns, regulatory control and certification. Online J Public Health Inform. 2014;5:1–23. 10.5210/ojphi.v5i3.4814 PMC3959919 24683442

[18] Hoare E, Milton K, Foster C, et al. Depression, psychological distress and Internet use among community-based Australian adolescents: a cross-sectional study. BMC Public Health. 2017;17:365. 28449667 10.1186/s12889-017-4272-1 PMC5406913

[19] Lattie EG, Adkins EC, Winquist N, et al. Digital mental health interventions for depression, anxiety, and enhancement of psychological well-being among college students: systematic review. J Med Internet Res. 2019;21:e12869. 31333198 10.2196/12869 PMC6681642

[20] Jere M, Augustine J. Investigating the factors that influence mobile phone adoption amongst the youth in South Africa. Mediterr J Soc Sci. 2014;5:519.

[21] Jesson J, Dietrich J, Beksinska M, et al. Food insecurity and depression: a cross-sectional study of a multi-site urban youth cohort in Durban and Soweto, South Africa. Trop Med Int Health. 2021 [cited 2021 Apr 5];26:687–700. 33666301 10.1111/tmi.13572

[22] StatsSA [Internet]. Pretoria, South Africa: department: statistics South Africa. Housebreaking still number one crime in SA; 2020 [cited 2021 Apr 6]. Available from: http://www.statssa.gov.za/?p=13811

[23] Hoare E, Milton K, Foster C, et al. The associations between sedentary behaviour and mental health among adolescents: a systematic review. Int J Behav Nutr Phys Act. 2016;13:108. 27717387 10.1186/s12966-016-0432-4 PMC5055671

[24] Pantic I, Damjanovic A, Todorovic J, et al. Association between online social networking and depression in high school students: behavioral physiology viewpoint. Psychiatr Danub. 2012;24:90–93. 22447092

[25] Gonzales AL, Hancock JT. Mirror, mirror on my Facebook wall: effects of exposure to Facebook on self-esteem. Cyberpsychol Behav Soc Netw. 2011;14:79–83. 21329447 10.1089/cyber.2009.0411

[26] Valenzuela S, Park N, Kee KF. Is there social capital in a social network site?: Facebook use and college students’ life satisfaction, trust, and participation. J Comput Mediat Commun. 2009;14:875–901.

[27] Steinfield C, Ellison NB, Lampe C. Social capital, self-esteem, and use of online social network sites: a longitudinal analysis. J Appl Dev Psychol. 2008;29:434–445.

[28] Simoncic TE, Kuhlman KR, Vargas I, et al. Facebook use and depressive symptomatology: investigating the role of neuroticism and extraversion in youth. Comput Human Behav. 2014;40:1–5. 25861155 10.1016/j.chb.2014.07.039 PMC4386284

[29] Essau CA, Lewinsohn PM, Seeley JR, et al. Gender differences in the developmental course of depression. J Affect Disord. 2010;127:185–190. 20573404 10.1016/j.jad.2010.05.016 PMC3754427

[30] Kessler RC, Aguilar-Gaxiola S, Alonso J, et al. The global burden of mental disorders: an update from the WHO World Mental Health (WMH) surveys. Epidemiol Psichiatr Soc. 2009;18:23–33. 19378696 10.1017/s1121189x00001421 PMC3039289

[31] Morrison CM, Gore H. The relationship between excessive internet use and depression: a questionnaire-based study of 1,319 young people and adults. Psychopathology. 2010;43:121–126. 20110764 10.1159/000277001

[32] Harris B, Regan T, Schueler J, et al. Problematic mobile phone and smartphone use scales: a systematic review. Front Psychol. 2020;11:672. 32431636 10.3389/fpsyg.2020.00672 PMC7214716

[33] Rikkers W, Lawrence D, Hafekost J, et al. Internet use and electronic gaming by children and adolescents with emotional and behavioural problems in Australia - results from the second child and adolescent survey of mental health and wellbeing. BMC Public Health. 2016;16:399. 27178325 10.1186/s12889-016-3058-1 PMC4866411

[34] Jenaro C, Flores N, Gómez-Vela M, et al. Problematic internet and cell-phone use: psychological, behavioral, and health correlates. Addict Res Theory. 2007;15:309–320.

[35] Jiang Z, Shi M. Prevalence and co-occurrence of compulsive buying, problematic Internet and mobile phone use in college students in Yantai, China: relevance of self-traits. BMC Public Health. 2016;16:1211. 27905905 10.1186/s12889-016-3884-1 PMC5131526

[36] Herman AA, Stein DJ, Seedat S, et al. The South African Stress and Health (SASH) study: 12-month and lifetime prevalence of common mental disorders. S Afr Med J. 2009;99:339–344. 19588796 PMC3191537

[37] Roser K, Schoeni A, Foerster M, et al. Problematic mobile phone use of Swiss adolescents: is it linked with mental health or behaviour? Int J Public Health. 2016;61:307–315. 26450576 10.1007/s00038-015-0751-2

[38] Wang C, Lee MK, Yang C, et al. Understanding problematic smartphone use and its characteristics: a perspective on behavioral addiction. In: Transforming healthcare through information systems: Heidelberg, Germany: springer. 2016. p. 215–225.

[39] Dietrich JJ, Laher F, Hornschuh S, et al. Investigating sociodemographic factors and HIV risk behaviors associated with social networking among adolescents in Soweto, South Africa: a cross-sectional survey. JMIR Public Health Surveill. 2016;2:e154. 27683173 10.2196/publichealth.4885 PMC5074647

[40] Porter G, Hampshire K, Abane A, et al. Intergenerational relations and the power of the cell phone: perspectives on young people’s phone usage in sub-Saharan Africa. Geoforum. 2015;64:37–46.

[41] Norris L, Swartz L, Tomlinson M. Mobile phone technology for improved mental health care in South Africa: possibilities and challenges. S Afr J Psychol. 2013;43:379–388.

[42] Sibeko G, Temmingh H, Mall S, et al. Improving adherence in mental health service users with severe mental illness in South Africa: a pilot randomized controlled trial of a treatment partner and text message intervention vs. treatment as usual. BMC Res Notes. 2017;10:584. 29121999 10.1186/s13104-017-2915-z PMC5679373

[43] Mall S, Sibeko G, Temmingh H, et al. Using a treatment partner and text messaging to improve adherence to psychotropic medication: a qualitative formative study of service users and caregivers in Cape Town, South Africa. Afr J Psychiatry (Johannesbg). 2013;16:364–370. 24051670 10.4314/ajpsy.v16i5.49

[44] North D, Johnston K, Ophoff J. The use of mobile phones by South African university students. IISIT. 2014;11:115–138.

[45] Chetty-Mhlanga S, Fuhrimann S, Eeftens M, et al. Different aspects of electronic media use, symptoms and neurocognitive outcomes of children and adolescents in the rural Western Cape region of South Africa. Environ Res. 2020;184:109315. 32172071 10.1016/j.envres.2020.109315

[46] Dietrich JJ, Coetzee J, Otwombe K, et al. Adolescent-friendly technologies as potential adjuncts for health promotion. Health Educ. 2014;114:304–318.

[47] Dietrich JJ, Hornschuh S, Khunwane M, et al. A mixed methods investigation of implementation barriers and facilitators to a daily mobile phone sexual risk assessment for young women in Soweto, South Africa. PLoS One. 2020;15:e0231086. 32324753 10.1371/journal.pone.0231086 PMC7179867

[48] Dietrich JJ, Lazarus E, Andrasik M, et al. Mobile phone questionnaires for sexual risk data collection among young women in Soweto, South Africa. AIDS Behav. 2018;22:2312–2321. 29594618 10.1007/s10461-018-2080-y PMC6176864

[49] Miller CL, Nkala B, Closson K, et al. The Botsha Bophelo adolescent health study: a profile of adolescents in Soweto, South Africa. SAJHIVMED. 2017;18:731. 10.4102/sajhivmed.v18i1.731 PMC5843033 29568638

[50] Buckley J, Otwombe K, Joyce C, et al. Mental health of adolescents in the era of antiretroviral therapy: is there a difference between HIV-infected and uninfected youth in South Africa? J Adolesc Health. 2020;67:76–83. 32269000 10.1016/j.jadohealth.2020.01.010

[51] Kaida A, Dietrich JJ, Laher F, et al. A high burden of asymptomatic genital tract infections undermines the syndromic management approach among adolescents and young adults in South Africa: implications for HIV prevention efforts. BMC Infect Dis. 2018;18:499. 30285705 10.1186/s12879-018-3380-6 PMC6171143

[52] Bradley KL, Bagnell AL, Brannen CL. Factorial validity of the Center for Epidemiological Studies Depression 10 in adolescents. Issues Ment Health Nurs. 2010;31:408–412. 20450343 10.3109/01612840903484105

[53] Baron EC, Davies T, Lund C. Validation of the 10-item centre for epidemiological studies depression scale (CES-D-10) in Zulu, Xhosa and Afrikaans populations in South Africa. BMC Psychiatry. 2017;17:6. 28068955 10.1186/s12888-016-1178-x PMC5223549

[54] Taber KS. The use of Cronbach’s alpha when developing and reporting research instruments in science education. Res Sci Edu. 2018;48:1273–1296.

[55] Bantjes J, Lochner C, Saal W, et al. Prevalence and sociodemographic correlates of common mental disorders among first-year university students in post-apartheid South Africa: implications for a public mental health approach to student wellness. BMC Public Health. 2019;19:922. 31291925 10.1186/s12889-019-7218-y PMC6617861

[56] Dowdall N, Ward CL, Lund C. The association between neighbourhood-level deprivation and depression: evidence from the South African national income dynamics study. BMC Psychiatry. 2017;17:395. 29228912 10.1186/s12888-017-1561-2 PMC5725901

[57] Silver S, Johnson C, [Internet]. Washington (DC): pew Research Center. Majorities in sub-Saharan Africa own mobile phones, but smartphone adoption is modest; 2018 Oct 9 [cited 2020 Dec 6]. Available from: https://www.pewresearch.org/global/2018/10/09/majorities-in-sub-saharan-africa-own-mobile-phones-but-smartphone-adoption-is-modest/

[58] Gilbert P, [Internet]. Johannesburg: ICASA. SA smartphone penetration now at over 80%, says ICASA; 2019 Apr 3 [cited 2020 Sep 1]. Available from: https://www.itweb.co.za/content/GxwQDM1AYy8MlPVo

[59] Seko Y, Kidd S, Wiljer D, et al. Youth mental health interventions via mobile phones: a scoping review. Cyberpsychol Behav Soc Netw. 2014;17:591–602. 25007383 10.1089/cyber.2014.0078

[60] Fedele DA, Cushing CC, Fritz A, et al. Mobile health interventions for improving health outcomes in youth: a meta-analysis. JAMA Pediatr. 2017;171:461–469. 28319239 10.1001/jamapediatrics.2017.0042 PMC6037338

[61] Lup K, Trub L, Instagram RL. #instasad?: exploring associations among Instagram use, depressive symptoms, negative social comparison, and strangers followed. Cyberpsychol Behav Soc Netw. 2015;18:247–252. 25965859 10.1089/cyber.2014.0560

[62] Burns JK, Tomita A, Lund C. Income inequality widens the existing income-related disparity in depression risk in post-apartheid South Africa: evidence from a nationally representative panel study. Health Place. 2017;45:10–16. 28237744 10.1016/j.healthplace.2017.02.005 PMC5438466

[63] WageIndicator, [Internet]. Amsterdam: wageIndicator Foundation. ARCHIVE- Living wage series - South Africa - September 2019 - In Rand, per Month; 2021 [cited 2021 Apr 6]. Available from: https://wageindicator.org/salary/living-wage/archive-no-index/south-africa-living-wage-series-september-2019

